# Resveratrol Supplementation in Obese Pregnant Rats Improves Maternal Metabolism and Prevents Increased Placental Oxidative Stress

**DOI:** 10.3390/antiox11101871

**Published:** 2022-09-21

**Authors:** Guadalupe L. Rodríguez-González, Lilia Vargas-Hernández, Luis A. Reyes-Castro, Carlos A. Ibáñez, Claudia J. Bautista, Consuelo Lomas-Soria, Nozomi Itani, Guadalupe Estrada-Gutierrez, Aurora Espejel-Nuñez, Arturo Flores-Pliego, Araceli Montoya-Estrada, Enrique Reyes-Muñoz, Paul D. Taylor, Peter W. Nathanielsz, Elena Zambrano

**Affiliations:** 1 Reproductive Biology Department, Instituto Nacional de Ciencias Médicas y Nutrición Salvador Zubirán, Mexico City 14080, Mexico; 2 Hospital de Ginecología y Obstetricia No. 4 Luis Castelazo Ayala, Mexico City 01090, Mexico; 3 CONACyT-Cátedras, Reproductive Biology Department, Instituto Nacional de Ciencias Médicas y Nutrición Salvador Zubirán, Mexico City 14080, Mexico; 4 Department of Women and Children’s Health, School of Life Course and Population Sciences, King’s College London and King’s Health Partners, London SE1 7EH, UK; 5 Research Direction, Instituto Nacional de Perinatología Isidro Espinosa de los Reyes, Mexico City 11000, Mexico; 6 Department of Immunobiochemistry, Instituto Nacional de Perinatología Isidro Espinosa de los Reyes, Mexico City 11000, Mexico; 7 Coordination of Gynecological and Perinatal Endocrinology, Instituto Nacional de Perinatología Isidro Espinosa de los Reyes, Mexico City 11000, Mexico; 8 Wyoming Center for Pregnancy and Life Course Health Research, Department of Animal Science, University of Wyoming, Laramie, WY 82071, USA

**Keywords:** maternal obesity, fetuses, liver, placenta, resveratrol, supplementation

## Abstract

Maternal obesity (MO) causes maternal and fetal oxidative stress (OS) and metabolic dysfunction. We investigated whether supplementing obese mothers with resveratrol improves maternal metabolic alterations and reduces OS in the placenta and maternal and fetal liver. From weaning through pregnancy female Wistar rats ate chow (C) or a high-fat diet (MO). One month before mating until 19 days’ gestation (dG), half the rats received 20 mg resveratrol/kg/d orally (Cres and MOres). At 19dG, maternal body weight, retroperitoneal fat adipocyte size, metabolic parameters, and OS biomarkers in the placenta and liver were determined. MO mothers showed higher body weight, triglycerides and leptin serum concentrations, insulin resistance (IR), decreased small and increased large adipocytes, liver fat accumulation, and hepatic upregulation of genes related to IR and inflammatory processes. Placenta, maternal and fetal liver OS biomarkers were augmented in MO. MOres mothers showed more small and fewer large adipocytes, lower triglycerides serum concentrations, IR and liver fat accumulation, downregulation of genes related to IR and inflammatory processes, and lowered OS in mothers, placentas, and female fetal liver. Maternal resveratrol supplementation in obese rats improves maternal metabolism and reduces placental and liver OS of mothers and fetuses in a sex-dependent manner.

## 1. Introduction

Obesity is currently an epidemic among women of reproductive age, in both developed and developing countries and has become one of the most important health issues during pregnancy increasing well-known risk factors for both the mother and her offspring [1,2]; these include changes in maternal glucose and lipid metabolism (especially predisposition to gestational diabetes), abnormal pregnancy hormone concentrations [3], stillbirth, premature birth, and macrosomia [3,4]. According to the developmental origins of health and disease hypothesis, offspring of obese mothers are at greater risk of developing obesity, diabetes, and cardiovascular disorders in adult life [5]. Several mechanisms have been proposed that link perinatal challenges with the development of diseases later in life, such as altered placental function, changes in the endocrine milieu, and epigenetic modifications [6,7]. However, oxidative stress, defined as an imbalance of pro-oxidants and antioxidants, is a key mechanism by which maternal obesity drives developmental programming, as the developing fetus is particularly vulnerable to oxidative stress due to its inadequate antioxidant defense mechanisms [8]. In addition, chronic inflammation is common in obese individuals, and is strongly linked to insulin resistance. Therefore, improving maternal antioxidant capacity as well as preventing obesity, insulin resistance, and type 2 diabetes during pregnancy can benefit fetal development and growth. Accordingly, it is important to establish preventive intervention strategies to improve the quality of life for both mother and offspring [9].

Resveratrol is a polyphenolic compound produced naturally by plants and fruits, such as blackberries, blueberries, and the skin of red grapes, which has been shown to have anti-obesogenic, anti-inflammatory and antioxidant properties [10,11,12] and thus could potentially exert protective effects in the setting of maternal obesity. In rodent models, resveratrol has been used as a therapeutic agent to treat pregnancy disorders such as gestational diabetes [13], preeclampsia [14] and fetal growth restriction [15]. In addition, it has been suggested that consuming resveratrol during pregnancy may help to prevent later-life diseases by protecting offspring from factors that increase susceptibility during developmental stages [16,17,18,19]. Studies in rats have demonstrated that maternal resveratrol supplementation during pregnancy improves oxidative stress biomarkers and metabolic dysfunction caused by a low-protein diet in the mother, placenta, and offspring [18] and lowers fetal oxidative stress and apoptosis in a streptozotocin-induced diabetic model [20,21]. In addition, it has been shown that maternal obesity leads to morphological changes in the small intestine as well as metabolic alterations in aged offspring, and that maternal resveratrol supplementation prior to and throughout pregnancy and lactation improved the offspring’s intestinal morphological changes and metabolic profiles in a sex-dependent manner [15].

Although there is growing evidence in animal models that support the benefits of maternal resveratrol supplementation during pregnancy in obese mothers, the mechanisms of resveratrol action are poorly understood. Furthermore, while many examples of developmental programming have been shown to be sexually dimorphic there is little evidence to determine if fetal responsiveness to resveratrol exposure differs according to sex. We aimed to investigate (1) the effects of resveratrol supplementation on maternal metabolic health and inflammatory and oxidative stress states of pregnant obese rats, and (2) any evidence for sex differences in fetal responses to resveratrol exposure to further understand the potential mechanisms underlying its programming effects in offspring health. We hypothesized that maternal resveratrol supplementation prior to and during pregnancy will reduce both maternal and fetal oxidative stress and improve metabolic health.

## 2. Materials and Methods

### 2.1. Standardization of Females Recruited for Breeding as Mothers

The Animal Experimentation Ethics Committee of the Instituto Nacional de Ciencias Médicas y Nutrición Salvador Zubirán (INCMNSZ), Mexico City, Mexico (ethical approval code, BRE-1868) approved all procedures, which are in accordance with the ARRIVE criteria for reporting animal studies [22,23]. Female albino Wistar rats were born and raised in the INCMNSZ animal facility, which is accredited by and follows the guidelines of the Association for Assessment and Accreditation of Laboratory Animal Care International (AAALAC). Rats were kept in temperature-controlled rooms (22–23 °C) with controlled lighting (lights on from 07:00 to 19:00 h) and fed standard laboratory chow diet (Zeigler Rodent RQ22-5, Gardners, PA, USA) containing 22.0% protein, 5.0% fat, 31.0% polysaccharide, 31.0% simple sugars, 4.0% fiber, 6.0% minerals and 1.0% vitamins (*w*/*w*), physiological fuel 4.0 kcal/g. At 14–16 weeks of age (weighing 200–240 g), females were randomly assigned to breed with non-litter mates of proven fertility. At delivery (day 0), litters that provided Founder Generation (F0) mothers were culled to ten pups, each with at least four females. At weaning (day 21), one female F0 pup from each litter was randomly assigned to either a maternal control (C, n = 18) group fed chow diet or a maternal obesity (MO, n = 14) group fed a high-fat diet [24] containing 23.5% protein, 20.0% lard, 5.0% corn oil fat, 20.2% polysaccharide, 20.2% simple sugars, 5.0% fiber, 5.0% mineral mix, 1.0% vitamin mix (*w*/*w*), physiological fuel 4.8 kcal/g. Food and water were provided ad libitum. The high-fat diet was developed at the INCMNSZ’s specialized dietary unit. To ensure homogeneity in the developmental programming challenge and maternal genetics to which offspring were exposed by F0 mothers, only one F0 female from the same litter was included in any experimental group.

At postnatal day (PND) 90, one month before mating and during pregnancy and lactation, half of the F0 females from each group (C and MO) were maintained on their assigned diet and received either vehicle or 20 mg resveratrol/kg/d (Harmony Flavors and Ingredients S.A. de C.V., Mexico) daily orally as an individual dose by pipette separate from the feed to generate two additional groups Cres and MOres. On PND 120, F0 female rats were mated with proven male breeders and the day on which sperms were found at the vaginal smear was considered as the beginning of pregnancy (day 0). At 19 days of gestation (dG), F0 females from each group were killed to obtain serum, maternal and fetal tissue.

### 2.2. Maternal (F0) and Fetal Tissue Collection at 19dG

At 19dG and after 4 h of fasting, F0 rats from all groups were weighed and euthanized under general anesthesia with isoflurane, followed by decapitation using a rodent guillotine (Thomas Scientific, Swedesboro, NJ, USA) by trained staff knowledgeable in the technique. Blood was drawn from the trunk, and serum was separated and stored at −70 °C until biochemical and hormonal analysis. The uterine horns were rapidly exposed through a midline abdominal incision, and the fetuses were decapitated and quickly extracted. Male and female placentas and livers from each litter were cleaned and pooled together as one sample for each sex. The gender of fetuses was determined by the presence of testes or ovaries. Fat depots from F0 rats were excised and weighed to calculate the adiposity index (AI). AI = total adipose tissue × 100/body weight (g). Livers from F0 rats were removed, cleaned, and weighed. All maternal blood and fetal tissues were immediately frozen in liquid nitrogen and stored at −70 °C for further analysis. The retroperitoneal fat pad was fixed for histological analysis. The data shown is based on the following number of mothers—C: n = 8, Cres: n = 10, MO: n = 7, MOres: n = 7.

### 2.3. F0 Biochemical and Hormonal Analysis

Glucose, cholesterol and, triglycerides, were determined enzymatically using the auto analyzer Synchron CX (Beckman Coulter, Brea, CA, USA). Leptin and insulin were determined using a radioimmunoassay kit from Millipore (Burlington, MA, USA), respectively. Homeostatic model assessment (HOMA) was calculated from HOMA = [glucose (mmol/L) × insulin (μU/mL)]/22.5 [25,26].

### 2.4. F0 Liver Fat Content and Triglycerides Content

Liver fat was extracted by a modified Folch technique [27]. Samples were homogenized with 2 mL of 0.9% NaCl and 5 mL of chloroform:methanol (2:1). Homogenate phases were separated by centrifugation (1500× *g* for 15 min at 4 °C) and the organic phase was evaporated under a stream of nitrogen and the extracted fat was weighed. The fat was re-suspended in a solution of isopropanol:triton (1:1000) to measure liver triglycerides content by using a RANDOX triglycerides kit, according to the manufacturer’s instructions (RANDOX, Crumlin, UK).

### 2.5. F0 Adipose Tissue Histology

The retroperitoneal fat pad samples were fixed in 10% paraformaldehyde-PBS 0.05 M and later dehydrated and paraffin-embedded. Sections of paraffin-embedded retroperitoneal fat with a thickness of 5 μm were mounted on poly-L-lysine-coated slides. After deparaffinization and rehydration, the slides were stained with hematoxylin and eosin [28]. Histology slides were examined under a light microscope Olympus BX51 (Melville, NY, USA) at 20 × magnification. Adipocyte size (AS) was measured manually by delimiting the adipocyte cross-sectional area in digital images using AxioVisio LE software real 4.8 version (Zeiss^®^ copyright 2006–2010 Stuttgart, Germany) in at least 229 cells per group corresponding to an average of 28 cells per rat. All histological measurements were performed by an observer blinded to the nature of the tissue source [29].

### 2.6. F0 Adipocyte Size (AS) Distribution

AS was measured as the cross-sectional area obtained in μm^2^. Histograms of the relative frequency of 500 μm^2^-area intervals were overlaid with their corresponding representative gamma distribution functions (GDFs). The AS distribution analysis was performed based on GDF modeling, as previously described in detail [30]. Briefly, GDFs were plotted using the shape [α = (Mean AS/AS Standard Deviation (ASSD))^2^] and scale [β = (ASSD^2^/ASMean)] parameter estimators and GAMMA.DIST function (Excel, Microsoft Office 365). Small and large adipocyte cut-off points were defined as the tenth and 90th percentile, respectively, from representative GDFs of C groups [30], using GAMMA.INV function (Excel, Microsoft Office 365). Extreme AS proportions for each rat was calculated in individual GDFs, as the cumulative probability below and above small and large AS cut-off points.

### 2.7. F0 Liver Gene Expression by Reverse Transcription Real-Time Quantitative Polymerase Chain Reaction (RT-qPCR)

Total RNA from the liver was isolated using 1 mL of TRIzol reagent (Invitrogen™ Waltham, MA, USA), with n = 6 independent random replicates in each group. The amount and quality of RNA were estimated spectrophotometrically at 260/280 nm and subsequently, a constant amount of RNA (3 µg) was reverse transcribed using a reverse transcription assay (Roche Diagnostics, Basel, Switzerland). All genes were subjected to the same qPCR conditions and were normalized to the housekeeping gene Rrpl32 as an internal control. The primer sequences used for qPCR are listed in Table 1. Amplifications were performed on a Light Cycler 2.0 real-time qPCR instrument (Roche) using Roche master mix and hydrolysis probes (Universal Probe Library, Roche) according to a standard protocol. Briefly, Taq DNA polymerase activation and denaturation were carried out at 95 °C for 10 min, followed by 45 amplification cycles of 10 s at 95 °C, 30 s at 60 °C, and 1 s at 72 °C. The qPCR data were analyzed using the ΔΔCT method [31].

### 2.8. Oxidative Stress Biomarkers in Liver and Placenta

Maternal and fetal liver and placenta were homogenized in saline at 4 °C and aliquots were frozen at −70 °C for subsequent protein quantification using the Bradford method and detection of oxidative stress biomarkers (reactive oxygen species and antioxidant enzymes). Lipid peroxidation was measured on the day of tissue homogenization. All determinations were made in duplicate, and results were averaged for statistical analysis.

#### 2.8.1. Lipoperoxidation Assay

To detect lipoperoxidation, malondialdehyde (MDA) was measured in 100 μL aliquots of serum, placenta, and liver homogenate using the thiobarbituric acid-reactive substances assay. All samples were read in a plate at 532 nm using a Perkin-Elmer LS50-B luminescence spectrometer. The results were expressed as nmol MDA/mg protein [18,24].

#### 2.8.2. Carbonylated Proteins

Protein extracts were obtained from the placenta and maternal and fetal liver (20 mg from each tissue) using the Tissue Protein Extraction (T-Per) reagent (78510, Thermo Scientific, Airport City, IL, USA), according to the manufacturer’s instructions. For the detection of carbonylated proteins, protein extracts were incubated with 2,4-dinitrophenylhydrazine (DNPH) 10 mM in HCl 2.5 M at room temperature for 1 h, avoiding the incidence of light. The samples were precipitated with 20% trichloroacetic acid (TCA) and centrifuged at 3000 rpm for 5 min at 4 °C. The resulting pellets were rinsed twice by centrifugation with 1 mL 5% TCA, washed by centrifugation with 2 mL ethanol:ethyl acetate (1:1) and solubilized in 0.5 mL of 6 M guanidine in 20 mM KH_2_PO_4_, pH 2.3. All samples were read at 370 nm. The carbonyl concentration was determined using the extinction molar coefficient ε = 22 M^−1^ cm^−1^ and expressed as pmol PC/mg protein [32].

#### 2.8.3. 8-Oxo-2′-Deoxyguanosine (8-oxo-dG) Quantification

Tissue fragments weighing approximately 20 mg from the placenta and maternal and fetal liver were processed for genomic DNA extraction and stored at −70 °C according to the manufacturer’s instructions (Wizard genomic DNA purification kit, Promega, Madison, WI, USA). 1 μg/μL of purified DNA was analyzed by ELISA to determine the concentration of 8-oxo-dG (HT 8-oxo-dG ELISA Kit II, R&D Systems, Minneapolis, MN, USA) following the manufacturer’s instructions. All samples were read at 450 nm. Results are expressed in 8-oxo-dG nM/ DNA (µg/µL).

#### 2.8.4. Reactive Oxygen Species (ROS) Assay

ROS formation in 5 μL of the homogenized liver was estimated using methods previously reported in detail [33]. A standard curve was obtained using increasing concentrations of 2′,7′-dichlorofluorescein (DCF) and incubated in parallel with the samples (37 °C for 60 min). At the end of the incubation period fluorescent signals at an excitation wavelength of 488 nm and an emission wavelength of 525 nm were recorded in a Perkin-Elmer LS50-B luminescence spectrometer. Results were expressed as nmoles of DCF formed per mg protein per minute [18,24].

### 2.9. Superoxide Dismutase (SOD) Activity

SOD activity was determined in 10 μL aliquots of placental and maternal and fetal liver homogenate with a RANSOD kit (RANDOX; Crumlin, UK) as previously reported [18,24]. A standard curve was obtained according to the manufacturer’s instructions. All samples were read in a plate at 505 nm in a Perkin-Elmer LS50-B luminescence spectrometer at 0, 30 s and 3 min at 37 °C. Results were expressed as activity units/mg protein.

### 2.10. Glutathione Peroxidase (GPx) Activity

GPx activity was determined in a 10 μL aliquot liver homogenate with the RANSEL kit (RANDOX; Crumlin, UK). All samples were read at 304 nm in a Perkin-Elmer LS50-B luminescence spectrometer at baseline, 1, 2 and 3 min at 37 °C. Results were expressed as milliunits/mg protein [18,24].

### 2.11. Immunohistochemistry (Nitrotyrosine, SOD and GPx)

5 µm paraffin sections of the right liver lobe were deparaffinized in xylene and rehydrated with ethanol in descending concentrations. Antigen retrieval was achieved by incubating the sections for 10 min in 0.01 M citrate solution. At room temperature, 10 percent (*v*/*v*) H_2_O –MeOH was used to inhibit endogenous peroxidase activity. To prevent nonspecific binding, the sections were pre-incubated for 1 h with a protein blocking buffer (Agilent Dako, Santa Clara, CA, USA) and then incubated overnight at 48 °C with the primary antibody, mouse monoclonal antibody anti-nitrotyrosine (MAB5404; Millipore, Burlington, MA, USA) at 1:200, mouse monoclonal anti-SOD-1 (B-1 sc-271014; Santa Cruz Biotechnology, Dallas, TX, USA) or anti-GPx-1/2 (D-12 sc-133152; Santa Cruz Biotechnology, Dallas, TX, USA) at 1:50. Primary antibodies were diluted with 0.4% Triton in potassium phosphate-buffered saline (KPBS). After three 5-min washes, the slides were incubated with the corresponding secondary antibody for two hours at 48 °C (all diluted 1:1000). After washing the sections, immunostaining was detected using 3,3′-diaminobenzidine (DAB; Sigma Aldrich, St. Louis, MO, USA) with nickel sulfate, which was then visualized and photographed using an Olympus BX51 light microscope and analyzed with ImageJ software (Image-Pro Plus Version 3.1, Media Cybernetics, Inc., Rockville, MD, USA). Incubations in the absence of the primary antibody were used as negative controls. The negative staining controls are not shown in the interest of space.

### 2.12. Statistical Analysis

Physiological, biochemical, hormonal, oxidative stress biomarkers (n = 7–10 per group) and gene expression (n = 6 per group) data are presented as mean ± SEM. For each litter, fetal tissues were pooled together as one sample for each sex. To assess the statistical differences within the maternal diet and resveratrol supplementation, data were analyzed using two-way multiple analysis of variance (ANOVA), followed by the Tukey test. Median AS were assessed by the Kruskall–Wallis test and the cumulative AS between groups were compared by the Kolmogorov–Smirnov test using GraphPad Prism 7.04 software. A statistical analysis was performed to determine if AS distribution and adipocyte size differed between C and MO. In the present study the AS distribution (Kolmogorov–Smirnov test) and the proportions of small (MO: 0.002 versus C: 10%, *p* < 0.001) and large (MO: 96.9 vs. C: 10%, *p* < 0.001) adipocytes (Mann-Whitney U test) were different between C and MO. Therefore, the effect of resveratrol supplementation on the proportions of small and large adipocytes between C and Cres and MO and MOres were analyze using the same statistical test; these analyses were performed using Sigma Plot 11.0 software. Statistically significant differences were defined as *p* < 0.05.

## 3. Results

### 3.1. F0 Body Weight Prior and during Pregnancy

**Figure 1:**
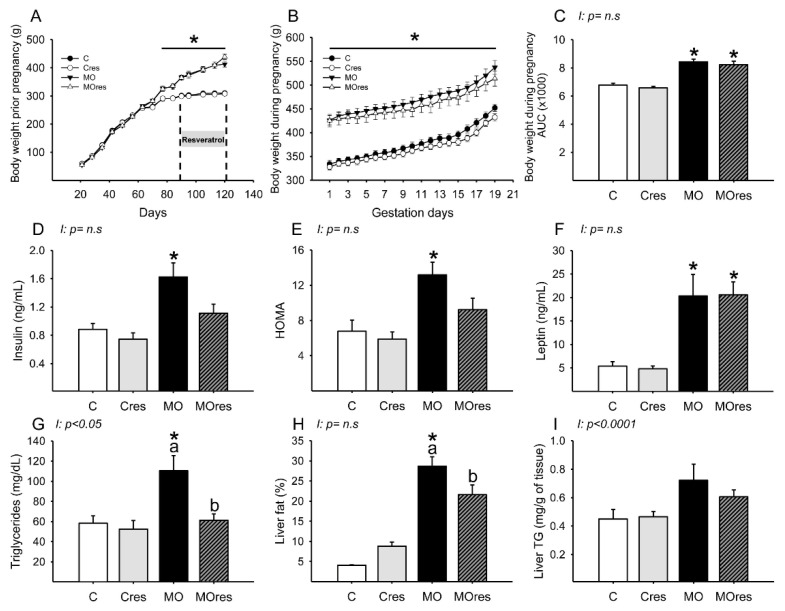
Maternal parameters: (**A**) body weight before pregnancy (g); (**B**) body weight during pregnancy (g); (**C**) body weight during pregnancy, area under the curve. Maternal metabolic parameters at 19dG: (**D**) Insulin (ng/mL); (**E**) HOMA; (**F**) Leptin (ng/mL); (**G**) Triglycerides (mg/dL); (**H**) Liver fat by folch (%); (**I**) Liver triglycerides (mg/g of tissue). Values are mean ± SEM, (C: control, n = 8; Cres: control + resveratrol, n = 10; MO: maternal obesity, n = 7; MOres: maternal obesity + resveratrol, n =7). Within the same group (C vs. Cres and MO vs. MOres) means labelled with different letters differ, *p* < 0.05; * different in comparison to the respective control (C vs. MO and Cres vs. MOres), *p* < 0.05. I = interaction between maternal diet and maternal resveratrol intervention, n.s. = not significant.

At weaning, the body weight of F0 females randomly assigned to be given either a control or a high-fat diet was similar in the C and MO groups but began to differ on day 70 of life. At the start of resveratrol supplementation and breeding time, the F0 females from the MO and MOres groups were heavier than the C and Cres groups (Figure 1A). MO and MOres mothers had a higher body weight at the beginning and throughout pregnancy than C and Cres groups (Figure 1B). In addition, the areas under the curve for maternal body weight were significantly increased in MO and MOres compared with C and Cres. No difference was observed between C vs. Cres and MO vs. MOres (Figure 1C).

#### Placenta and Fetal Weight at 19dG

There were no differences in male and female placenta weights among groups (Male: 0.59 ± 0.03, Cres: 0.62 ± 0.03, MO: 0.51 ± 0.04, MOres: 0.50 ± 0.03 g; Female: C: 0.59 ± 0.02, Cres: 0.57 ± 0.02, MO: 0.54 ± 0.05, MOres: 0.49 ± 0.02 g). Fetal weights were similar in all groups for both sexes (Male: 2.6 ± 0.1, Cres: 2.8 ± 0.1, MO: 2.0 ± 0.08, MOres: 2.3 ± 0.08 g; Female: C: 2.6 ± 0.09, Cres: 2.6 ± 0.11, MO: 2.0 ± 0.07, MOres: 2.2 ± 0.05 g).

### 3.2. F0 Food and Calorie Intake during Pregnancy

Based on averaged data, MO and MOres groups consumed less food per day during pregnancy than C and Cres (C: 27 ± 0.6, Cres: 26 ± 0.7, MO: 21 ± 0.5 *, MOres: 20 ± 0.7 * g/day; * different in comparison to the respective control (MO vs. C and MOres vs. Cres), *p* < 0.05), the calorie intake was similar in all groups (C: 108 ± 2.4, Cres: 102 ± 2.8, MO: 104 ± 2.5, MOres: 100 ± 3.4 Kcal/day).

### 3.3. F0 Body Weight and Fat Distribution at 19dG

Table 2 shows that MO and MOres groups had higher body weight, total fat, adiposity index, and retroperitoneal, omental, parametrial and periovaric fat than C and Cres groups. Mediastinal fat was similar in all groups.

### 3.4. F0 Metabolic Parameters at 19dG

Maternal glucose (C: 61 ± 9, Cres: 63 ± 6, MO: 65 ± 4, MOres: 66 ± 6 mg/dL) and cholesterol (C: 53 ± 4, Cres: 54 ± 5, MO: 54 ± 4, MOres: 48 ± 3 mg/dL) serum concentrations were similar in all experimental groups. MO had a higher insulin serum concentration and HOMA index than C, while the MOres group had similar levels to MO and Cres. (Figure 1D,E). Leptin serum concentrations were higher in both MO and MOres compared to C and Cres (Figure 1F). F0 from the MO group had greater serum triglyceride concentrations and a higher percentage of fat in the liver, but maternal resveratrol supplementation significantly reduced these parameters in MOres compared to MO (Figure 1G,H). Liver triglyceride content was similar in all groups (Figure 1I). Both triglycerides serum concentrations (*p* < 0.05) and liver triglycerides content (*p* < 0.0001) showed interaction between maternal diet and resveratrol supplementation.

### 3.5. F0 Adipocyte Size (AS) Distribution

**Figure 2:**
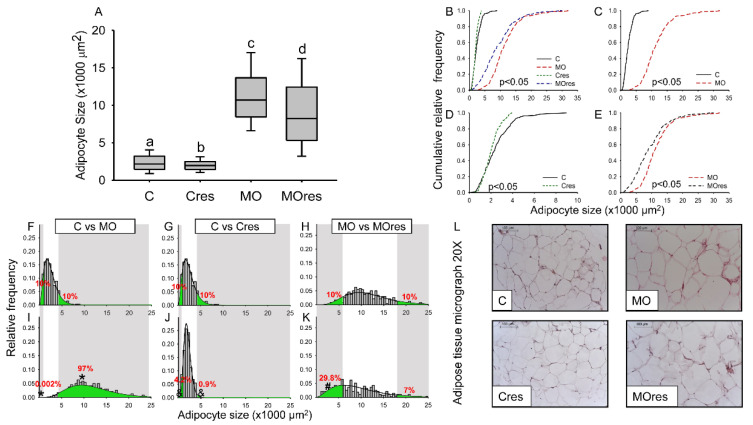
Maternal adipocyte characteristics at 19dG. (**A**) adipocyte size (×1000 μm^2^); (**B**–**E**) Cumulative relative frequency; (**F**–**K**) Relative frequency histograms and the gamma distribution function; (**L**) Representative micrograph with H&E (20×). Median adipocyte size labelled with different letters differ (*p* < 0.05) by Kruskal-Wallis tests. Statistical differences between adipocyte size distributions (*p* < 0.05) by two sample Kolmogorov–Smirnov test. Relative frequency by Mann-Whitney Rank Sum Test. Small and large adipocyte cut-off points are defined by the 10th and 90th percentile, respectively, of the C or MO group modeled by the gamma distribution. (C: control, n = 8; Cres: control + resveratrol, n = 10; MO: maternal obesity, n = 7; MOres: maternal obesity + resveratrol, n = 7). * *p* < 0.05 C vs. MO; & *p* < 0.05 C vs. Cres, # *p* < 0.05 MO vs. MOres, by non-parametric comparisons of the small and large adipocyte proportions.

Median AS was higher in F0 from the MO group than C, whereas maternal resveratrol supplementation significantly reduced the median AS in both Cres and MOres compared to C and MO, respectively (Figure 2A). In all groups, the cumulative frequency of AS distribution was different (Figure 2B). Figure 2C shows that the cumulative AS distribution in MO was wider than in C, but maternal resveratrol supplementation reduced the spread of the cumulative AS distribution in both Cres and MOres in comparison to C and MO (Figure 2D,E). Figure 2F,I show that the proportions of small (MO: 0.002 versus C: 10%, *p* < 0.001) and large (MO: 97% vs. C: 10%, *p* < 0.001) adipocytes differed between the MO and C groups. In the Cres F0 group, maternal resveratrol supplementation reduced the proportion of small and large adipocytes compared to C (Figure 2G,J). However, in MOres, maternal resveratrol supplementation increased the proportion of small adipocytes (Figure 2H,K). A representative micrograph of retroperitoneal adipose tissue from each group used to analyze the F0 AS distribution is shown in Figure 2L.

### 3.6. F0 Expression of Hepatic Metabolic and Inflammatory Genes

**Figure 3:**
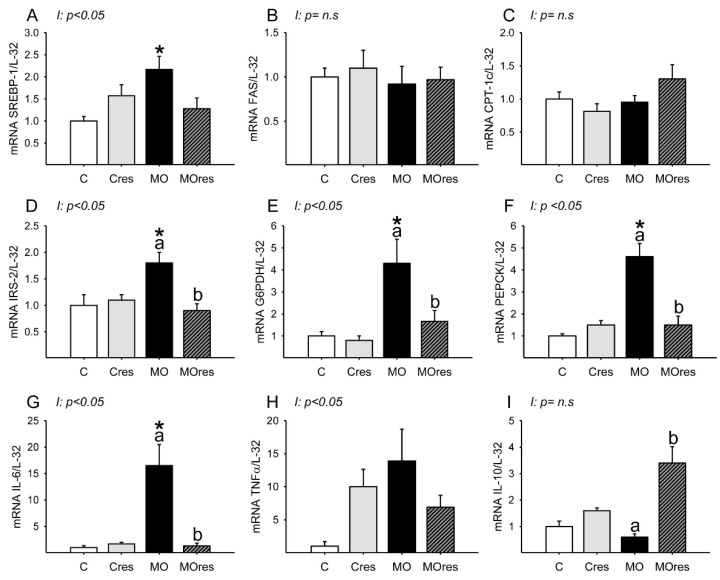
Maternal lipid and glucose metabolism and inflammatory gene expression in the liver at 19dG. (**A**) SREBP; (**B**) FAS; (**C**) CPT-1; (**D**) IRS-2; (**E**) G6PDH; (**F**) PEPCK; (**G**) IL-6; (**H**) TNF-α; (**I**) IL-10. Values are mean ± SEM, (C: control, n = 6; Cres: control + resveratrol, n = 6; MO: maternal obesity, n = 6; MOres: maternal obesity + resveratrol, n =6). Within the same group (C vs. Cres and MO vs. MOres) means labelled with different letters differ, *p* < 0.05; * different in comparison to the respective control (C vs. MO and Cres vs. MOres), *p* < 0.05. I = interaction between maternal diet and maternal resveratrol intervention, n.s. = not significant.

Messenger RNA expression of SREBP-1 was higher in MO than in C, and maternal resveratrol supplementation had no effect (Figure 3A). Hepatic FAS and CPT-1 messenger RNA expression were similar is all groups (Figure 3B,C). The MO group had higher hepatic messenger RNA expression of IRS-2, G6PDH, PEPCK than the C group, whereas maternal resveratrol supplementation decreased IRS-2, G6PDH and PEPCK mRNA expression in the livers of the MOres group compared to the MO group (Figure 3D–F). IL-6 messenger RNA expression was higher in MO than in C, and maternal resveratrol supplementation reduced IL-6 expression in MOres compared to MO (Figure 3G). Messenger RNA expression of IL-10 was similar in MO and C, but maternal resveratrol supplementation increased IL-10 expression in MOres when compared to MO (Figure 3I). Hepatic TNF-α expression was similar in all groups (Figure 3H). SREBP, IRS-2, G6PDH, PEPCK IL-6 and TNF-α expression showed an interaction between maternal diet and resveratrol supplementation, *p* < 0.05.

### 3.7. F0 Hepatic Oxidative Stress Biomarkers, Antioxidant Enzyme Activity and Gene Expression at 19dG

**Figure 4:**
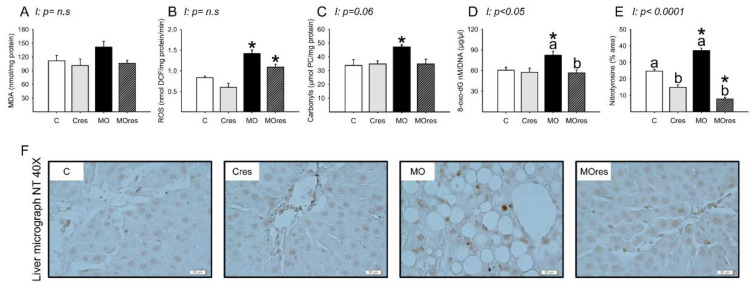
Maternal oxidative stress biomarkers in the liver at 19dG. (**A**) MDA (nmol/mg protein); (**B**) ROS (nmol DCF/mg protein/min); (**C**) Carbonyls (μmol PC/mg protein); (**D**) 8-oxo-dG (8-oxo-dG nM/DNA (μg/μL)); (**E**) Nitrotyrosine inmunostained area (%); (**F**) Representative micrograph of NT (40×). Values are mean ± SEM, (C: control, n = 8; Cres: control + resveratrol, n = 10; MO: maternal obesity, n = 7; MOres: maternal obesity + resveratrol, n = 7). Within the same group (C vs. Cres and MO vs. MOres) means labelled with different letters differ, *p* < 0.05; * different in comparison to the respective control (MO vs. C and MOres vs. Cres), *p* < 0.05. I = interaction between maternal diet and maternal resveratrol intervention, n.s. = not significant.

The MO group had higher serum MDA concentrations than the C group, whereas maternal resveratrol supplementation decreased serum MDA concentrations in the MOres group compared to MO (C: 423.5 ± 5.2, Cres: 449.1 ± 15.8, MO: 523.8 ± 28.5a *, MOres: 416.7 ± 14.9b nmol/100 μL serum; within the same group means labelled with different letters differ (MO vs. MOres), *p* < 0.05; * different in comparison to the respective control (MO vs. C), *p* < 0.05; interaction between maternal diet and resveratrol supplementation, *p* < 0.05). MDA concentrations in the liver were similar across groups (Figure 4A). ROS concentrations and the amount of protein carbonyl groups in MO livers were higher in MO than C, and maternal resveratrol supplementation had no effect on these parameters (Figure 4B,C). F0 livers from the MO group had elevated amounts of 8-oxo-dG compared with C and maternal resveratrol supplementation significantly reduced DNA oxidation in MOres compared to MO (Figure 4D). Livers from the MO group had a higher percentage of nitrotyrosine immunostained area than the C group, and maternal resveratrol supplementation reduced nitrotyrosine in both Cres and MOres compared to C and MO, respectively (Figure 4E). Figure 4F shows representative micrographs for liver nitrotyrosine immunostaining. 8-oxo-dG (*p* < 0.05), and nitrotyrosine (*p* < 0.0001) showed an interaction between maternal diet and resveratrol supplementation.

**Figure 5:**
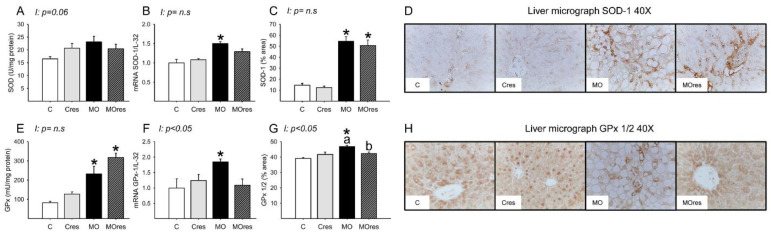
Maternal antioxidant enzyme activity and gene expression in the liver at 19dG. (**A**) SOD activity (U/mg protein); (**B**) SOD gene expression; (**C**) SOD inmunostained area (%); (**D**) Representative micrograph of SOD-1 (40×); (**E**) GPx activity (mU/mg protein); (**F**) GPx gene expression; (**G**) GPx 1/2 inmunostained area (%); (**H**) Representative micrograph of GPx 1/2 (40×). Values are mean ± SEM, (C: control, n = 8; Cres: control + resveratrol, n = 10; MO: maternal obesity, n = 7; MOres: maternal obesity + resveratrol, n = 7). Within the same group (C vs. Cres and MO vs. MOres) means labelled with different letters differ, *p* < 0.05; * different in comparison to the respective control (MO vs. C and MOres vs. Cres), *p* < 0.05. I = interaction between maternal diet and maternal resveratrol intervention, n.s. = not significant.

The MO group had higher hepatic messenger RNA expression of Nrf2, whereas maternal resveratrol supplementation decreased Nrf2 expression in the MOres group compared to MO (C: 1 ± 0.3, Cres: 0.8 ± 0.2, MO: 2.4 ± 0.5a*, MOres: 0.9 ± 0.1b; within the same group means labelled with different letters differ (MO vs. MOres), *p* < 0.05; * different in comparison to the respective control (MO vs. C), *p* < 0.05; interaction between maternal diet and resveratrol supplementation, *p* = 0.051). SOD activity was similar across groups (Figure 5A), while GPx activity were higher in MO than C, and maternal resveratrol supplementation had no effect on this parameter (Figure 5E). The hepatic messenger RNA expression of SOD-1, and GPx-1 was higher in MO as compared to the C group and maternal resveratrol supplementation does not decrease SOD-1, or GPx-1 in MOres (Figure 5B,F). MO livers had a higher percentage area of tissue immunostained for SOD and GPx than C. Maternal resveratrol supplementation reduced GPx in MOres when compared to MO but not in Cres compared with C (Figure 5C,G). Representative micrographs for liver SOD and GPx immunostaining are shown in Figure 5D,H, respectively. GPx% of area and expression showed an interaction between maternal diet and resveratrol supplementation, *p* < 0.05.

### 3.8. Placental Oxidative Stress Biomarkers and Antioxidant Enzyme Activity at 19dG

#### 3.8.1. Male Placenta

**Figure 6:**
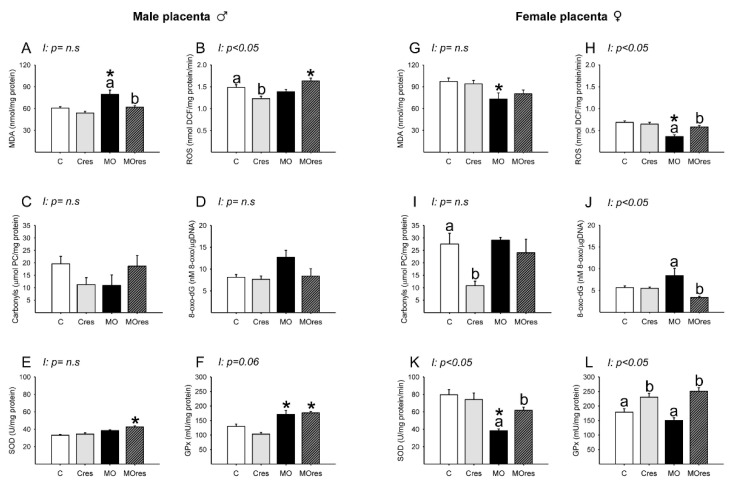
Oxidative stress biomarkers and antioxidant enzyme activity in the male and female placenta at 19dG. Male and female: (**A**,**G**) MDA concentration (nmol/mg protein); (**B**,**H**) ROS concentration (nmol DCF/mg protein/min); (**C**,**I**) Carbonyls concentration (μmol PC/mg protein); (**D**,**J**) 8-oxo-dG concentration (8-oxo-dG nM/DNA (μg/μL)); (**E**,**K**) SOD activity (U/mg protein); (**F**,**L**) GPx activity (mU/mg protein). Values are mean ± SEM, (C: control, n = 8; Cres: control + resveratrol, n = 10; MO: maternal obesity, n = 7; MOres: maternal obesity + resveratrol, n = 7). Within the same group (C vs. Cres and MO vs. MOres), means labeled with different letters differ, *p* < 0.05; * different in comparison to the respective control (MO vs. C and MOres vs. Cres), *p* < 0.05. I = interaction between maternal diet and maternal resveratrol supplementation, n.s. = not significant.

Placental lipoperoxidation evaluated by MDA concentration was increased in the MO group compared to C, and maternal resveratrol supplementation significantly reduced MDA levels in MOres when compared to MO (Figure 6A). The concentrations of ROS in C and MO were similar. However, ROS concentrations were reduced in Cres compared to C and increased in MOres compared to Cres (Figure 6B). The amount of carbonyl groups and 8-oxo-dG were similar across groups (Figure 6C,D). SOD activity was higher in MOres than Cres, while GPx activity was higher in both MO and MOres groups compared to C and Cres (Figure 6E,F). ROS concentrations showed an interaction between maternal diet and resveratrol supplementation, *p* < 0.05.

#### 3.8.2. Female Placenta

MDA concentrations were similar in MO and C groups (Figure 6G). ROS concentrations were lower in MO than C, and maternal resveratrol supplementation increased ROS levels in MOres compared to MO (Figure 6H). Maternal resveratrol supplementation decreased carbonyl groups in Cres compared to C and 8-oxo-dG in MOres compared to MO (Figure 6I,J). SOD activity was lower in MO than in C, and maternal resveratrol supplementation significantly increased SOD activity in MOres when compared to MO (Figure 6K). Cres and MOres had higher GPx activity than C and MO, respectively (Figure 6L). ROS and 8-oxo-dG levels as well as SOD and GPx activities, showed an interaction between a maternal diet and resveratrol supplementation, *p* < 0.05.

### 3.9. Fetal Hepatic Oxidative Stress Biomarkers and Antioxidant Enzyme Activity at 19dG

#### 3.9.1. Male Liver

**Figure 7:**
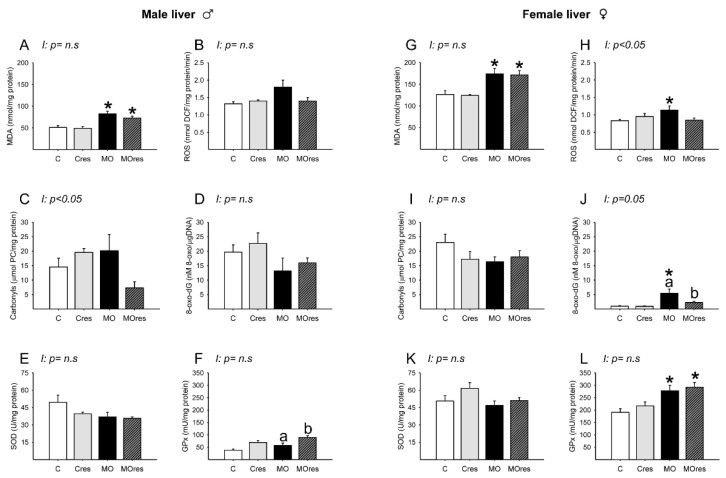
Oxidative stress biomarkers and antioxidant enzyme activity in male and female liver at 19dG. Male and female: (**A**,**G**) MDA concentration (nmol/mg protein); (**B**,**H**) ROS concentration (nmol DCF/mg protein/min); (**C**,**I**) Carbonyls concentration (μmol PC/mg protein); (**D**,**J**) 8-oxo-dG concentration (8-oxo-dG nM/DNA (μg/μL)); (**E**,**K**) SOD activity (U/mg protein); (**F**,**L**) GPx activity (mU/mg protein). Values are mean ± SEM, (C: control, n = 8; Cres: control + resveratrol, n = 10; MO: maternal obesity, n = 7; MOres: maternal obesity + resveratrol, n = 7). Within the same group (C vs. Cres and MO vs. MOres) means labelled with different letters differ, *p* < 0.05; * different in comparison to the respective control (MO vs. C and MOres vs. Cres), *p* < 0.05. I = interaction between maternal diet and maternal resveratrol supplementation, n.s. = not significant.

MDA concentrations in MO and MOres were higher compared to C and Cres, respectively (Figure 7A). The amounts of ROS, carbonyl groups, and 8-oxo-dG, as well as SOD activity, were similar across groups (Figure 7B–E). GPx activity was higher in MOres than in MO (Figure 7F). Carbonyls concentrations showed an interaction between maternal diet and resveratrol supplementation, *p* < 0.05.

#### 3.9.2. Female Liver

MDA concentrations and GPx activity in MO and MOres were higher compared to C and Cres, respectively (Figure 7G,L). ROS concentrations were higher in MO than in C (Figure 7H). The amounts of 8-oxo-dG in MO were higher than in C, and maternal resveratrol supplementation reduced 8-oxo-dG in MOres compared to MO (Figure 7J). The amounts of carbonyl groups and SOD activity were similar across groups (Figure 7I,K). ROS and 8-oxo-dG levels showed an interaction between maternal diet and resveratrol supplementation, *p* < 0.05.

## 4. Discussion

Normal pregnancy causes metabolic changes needed for fetal growth and development. Obesity and high-fat diets affect the mother’s physiology [34] and increases offspring susceptibility to metabolic and cardiovascular illness [24,35,36]. There is an urgent need to develop intervention strategies to explore potential mechanisms involved in fetal programming, to evaluate sexual dimorphism and control the negative effects of maternal obesity and its associated metabolic comorbidities on offspring health. Diet and exercise have been widely used to prevent and treat abnormal metabolism in obese mothers during pregnancy [9,26,30,37,38]. However, it is not always possible to implement these interventions. The consumption of natural bioactive compounds has been linked to the prevention of chronic diseases, and they work through a variety of metabolic, cellular, and molecular mechanisms [39]. Resveratrol is a polyphenolic compound with anti-obesogenic, anti-diabetic, and antioxidant effects [19]. However, the influence of resveratrol supplementation prior to and during pregnancy on fetal responsiveness to early resveratrol exposure and the potential mechanisms involved have not been thoroughly explored. In the present study, we observed that maternal resveratrol supplementation in obese pregnant rats lessened maternal metabolic dysfunction and lowered oxidative stress in mothers, placentas, and fetal liver in a sex-dependent manner. Importantly, no adverse effects were observed when pregnant control rats were supplemented with resveratrol.

Maternal obesity is caused by dysregulated energy homeostasis, which is induced by overeating fat-, calorie-, and sugar-rich diets [40]. We have reported that a high-fat diet before pregnancy until the end of lactation increases MO mothers’ body weight, retroperitoneal, and total body fat [26,38,41]. The present study demonstrates the reproducibility of our model by observing the same maternal phenotype. Clinical trials have reported the effects of resveratrol consumption on obesity; however, the results differ, with some studies suggesting a reduction in body weight [42] and others demonstrating no effect on body weight but metabolic benefits [43,44]. In animal studies, giving resveratrol to pregnant rats reduced the negative metabolic effects of gestational diabetes or obesity on both mothers and offspring [45,46]. Our findings showed that although MOres mothers do not display a decrease in body weight, the metabolic alterations are reduced. MO and MOres mothers at 19dG showed a reduction in food intake without changes in energy intake. A potential explanation is that the maternal high-fat diet is known to decrease appetite [47].

White adipose tissue regulates energy homeostasis by acting as a caloric reservoir, and it expands through hyperplasia (increase in adipocyte number) and/or hypertrophy (increase in adipocyte size) [48,49]. Adipocyte hyperplasia is associated with the healthy expansion of white adipose tissue via the proliferation and differentiation of adipocyte precursor cells [50,51]. In obese individuals, adipose tissue expansion and storage capacity become dysfunctional [48,49] due to hypertrophy caused by the growth of existing adipocytes, macrophage infiltration, and fibrosis [50,51]. When adipose tissue capacity is exceeded, fat is stored as triacylglycerols in peripheral organs. An overabundance of lipids produces toxic reactive lipid species that accumulate in metabolically essential organs such as the pancreas, liver, and heart; this is called lipotoxicity, and it can lead to insulin resistance, heart failure, and liver steatosis [52]. In the present study, the retroperitoneal fat of MO mothers showed a reduction in small adipocytes and an increase in large adipocytes. Therefore, the reduction of the small and the increase in the large adipocytes observed in the MO group may be associated with increased insulin serum levels and HOMA index as well as hypertriglyceridemia and liver fat accumulation. In an in vitro study on 3T3-L1 pre-adipocytes, resveratrol reduced lipid accumulation in a dose-dependent manner. Additionally, in 3T3-L1 adipocytes, the analysis of the expression of SREBP-1c, PPARγ, and C/EBPα revealed that resveratrol decreased both gene expression and protein abundance of these transcription factors; these resveratrol-induced changes are likely to modulate adipogenesis [53]. In our model, maternal resveratrol supplementation in the Cres group decreased lipid accumulation in conjunction with a reduction in the number of small and large adipocytes, whereas in the MOres group liver fat accumulation was reduced and white adipose tissue expansion changed from hypertrophic to hyperplasic, as shown by a decrease in the number of large adipocytes and an increase in the number of small adipocytes.

Insulin resistance is recognized as a key pathophysiological factor in nonalcoholic fatty liver disease (NAFLD). The initial explanation for its etiology was the “two-hit theory”, in which the first hit was characterized by an accumulation of fatty acids and triglycerides in the liver; while the second hit consisted of chronic stresses such as increased lipid peroxidation, formation of ROS, and increased pro-inflammatory responses [54]. Three sources provide fatty acids to hepatic triglycerides: dietary fat, free fatty acids released from adipose tissue, and hepatic de novo lipogenesis [55]; the latter drives NAFLD in insulin resistance [56]. SREBP-1c is a key transcription factor that controls hepatic de novo lipogenesis in response to insulin [57]. In a diabetic mouse model, it has been shown that elevated nuclear SREBP-1c levels and hyperinsulinemia contribute to the elevated rates of hepatic fatty acid production, leading to steatosis [58]. Another study reported that resveratrol administration improved lipid metabolism, decreased NAFLD and the expression of genes related to adipogenesis in the liver of mice fed an obesity-inducing diet [59]. In our model, MO mothers exhibited a rise in hepatic SREBP expression, insulin serum concentration, HOMA index, and liver fat accumulation. Maternal resveratrol supplementation ameliorated all these adverse outcomes.

The liver maintains glucose and energy homeostasis. IRS-1 and IRS-2 regulate insulin-dependent hepatic glucose metabolism, and their dysregulation contributes to insulin resistance and type 2 diabetes. IRS-1 causes insulin resistance, while IRS-2 is needed for hepatic insulin activity [60], and its increase is related to prediabetes [61]. Increased insulin signaling may enhance hepatic lipogenesis by activating SREBP-1c, which stimulates fatty acid synthesis-related genes [62]. Our results showed that in obese mothers, there was an increase in the hepatic expression of SREBP-1, IRS-2, and the HOMA index. Thus, maternal obesity, together with a high-fat diet consumption prior to and during pregnancy, can increase insulin resistance in the liver and stimulate fatty acid synthesis via insulin signal transduction activation. Resveratrol supplementation during pregnancy reduced the upregulation of SREBP-1, IRS-2, and HOMA index, suggesting an increase in insulin sensitivity.

G6PDH is a key enzyme that shunts from the glycolytic pathway, catalyzes ribose synthesis for nucleic acid production, and generates cytosolic NADPH, an important cofactor in metabolic pathways like fatty acid and cholesterol biosynthesis [63,64]. In addition, G6PDH regulates cellular oxidation by providing NADPH to ROS-producing and-scavenging enzymes [65]. Under pathological conditions such as obesity and atherosclerosis, G6PDH enhances cellular ROS formation and pro-inflammatory signaling through increased availability of NADPH to ROS-producing enzymes [66]. G6PDH has been found to be elevated in the liver of Zucker rats with type 2 diabetes and in adipocytes of both genetic (db/db and ob/ob) and diet-induced obese rats [67,68]. Additionally, the overexpression of G6PDH has been associated to changes in lipid metabolism, adipocytokine expression, and insulin-resistant adipocytes [69]. We observed that resveratrol supplementation reduced the increase in triglyceride serum concentrations and the overexpression of G6PDH that was caused by maternal obesity.

PEPCK is a key enzyme that regulates gluconeogenesis and glycogenolysis, and it is highly regulated by the glucagon-insulin axis [70,71]. In mice, it has been shown that hepatic PEPCK gene modification has profound effects on systemic glucose metabolism. Two-fold overexpression of PEPCK in transgenic mice causes insulin resistance [72], whereas seven-fold overexpression causes hyperglycemia [73]. In a different rat study, a high-fat-high-sugar diet caused an increase in serum lipids and glucose concentrations as well as negative effects on liver and renal function and an increase in PEPCK expression. Furthermore, PEPCK enzyme activity and glycogenolysis in the liver were downregulated by oxytocin therapy [74]. In our model, obese mothers showed insulin resistance and overexpression of hepatic PEPCK, which were improved by resveratrol supplementation.

Normal-weight and obese pregnancies differ in numerous physiological ways. Women with normal-weight pregnancies have decreased insulin sensitivity, increased protein synthesis, and increased lipogenesis and fat accretion, while obese pregnant women are more likely to develop gestational diabetes [75] and to have increased elevations in pro-inflammatory cytokines, glucose, lipids, and amino acids [76,77], exposing the growing placenta and fetus to a mix of inflammatory cytokines and excess nutrition in utero, which may have long-lasting effects on postnatal life.

IL-6 is a pro-inflammatory cytokine that is secreted by a variety of organs, including hepatocytes and adipose tissue [78]; it has been reported that obesity and NAFLD are related to IL-6 hepatic overexpression [79]. Large population studies have reported elevated IL-6 levels as a predictor of diabetes risk [80]. In addition, there is a link between maternal IL-6 concentrations at term and newborn fat mass, although there was no correlation between cord blood IL-6 and neonatal fat mass. In a study involving obese pregnant women, adipocyte hypertrophy was associated with a greater degree of inflammation [81]; this indicated that maternal inflammation might be responsible for the elevated risk of offspring adiposity [82]. IL-10, on the other hand, is an anti-inflammatory cytokine that attenuates the inflammatory processes [78], and it has been suggested to be protective against the development of diabetes and metabolic syndrome [83]. In macrophages activated by lipopolysaccharide, IL-10 inhibited the production of the cytokines TNFα and IL-6 [84]. Our data showed that obese mothers exhibited metabolic inflammation as observed by the increase in hypertrophic adipocytes and HOMA index as well as for the overexpression of IL-6 and the reduced expression of IL-10, which was prevented by maternal resveratrol supplementation.

Oxidative stress occurs when there is an imbalance of free radicals and antioxidants [85]. Moderate amounts of ROS are essential for the maintenance of numerous physiological functions; nevertheless, excessive ROS production has deleterious effects on a variety of cellular components, including proteins, lipids, and DNA. Increased MDA, carbonylated proteins, and 8-oxo-dG are markers of lipid peroxidation, protein oxidation, and DNA oxidation, respectively [86]. Obesity can produce systemic oxidative stress by a variety of biochemical processes, including superoxide production from NADPH oxidases, oxidative phosphorylation, and protein kinase C activation. Other factors that also contribute to oxidative stress in obesity include hyperleptinemia, low antioxidant defense, chronic inflammation, type of diet and mitochondrial dysfunction [87]. During a normal pregnancy, antioxidants are crucial for sustaining cellular function. However, in pathological situations, the redox equilibrium is disrupted [88]. In the early stages of obesity, antioxidant enzymes are upregulated to prevent oxidative damage; but, as fat accumulates, the antioxidant defense is overwhelmed [89]. Nrf2 is a transcription factor that is essential for the maintenance of redox and metabolic balance by regulating cellular antioxidants and reducing inflammatory stress. Activation of Nrf2 may have a protective role against oxidative stress for disorders associated with chronic inflammation and ROS generation [90]. In our model, maternal obesity and a high-fat diet led to adipocyte hypertrophy in the retroperitoneal fat, an increase in insulin and the HOMA index, liver steatosis, and an increase in biomarkers of oxidative stress and antioxidant enzymes, as well as activation of GPx and Nrf2. Resveratrol supplementation of obese mothers improved these variables.

Fetal responses to stress factors show sexual dimorphism. During preimplantation, male and female embryos have significant transcriptional dimorphism, which is mostly caused by incomplete X chromosome inactivation; later in development, gonadal formation results in hormonal variations between sexes [91]. Male and female placentas and fetuses respond differently to the intrauterine environment [92]; for example, under adverse intrauterine conditions, females exhibit better placental adaptation and fetal growth [93]. Thus, it has been reported that during perinatal mouse lung development, females show higher GPx1 mRNA transcription levels than males, suggesting females may be better protected against oxidative stress [94]. In support of this view, mice fed a moderate trans-fat diet the hepatic expression of lipogenic, and antioxidant proteins were higher in female than in male mice [95]. In our results, it is evident that the placentas and the fetal liver of males and females in the control group differ in the concentrations of oxidative stress biomarkers and the activity of antioxidant enzymes, and that both the placenta and fetal liver of females from obese mothers exhibited a greater response than males to counteract the harmful effects of the intrauterine environment to which they are exposed. Finally, we observed that resveratrol supplementation in the Cres group reduced male placental ROS concentrations; therefore, the consumption of resveratrol during normal pregnancy should be thoroughly investigated [96]. Moreover, ROS play a crucial role as secondary messengers in numerous intracellular signaling cascades and mediate essential cellular activities including proliferation, differentiation, and death [97]. Consequently, the reduced concentrations observed in female MO placentas may have a negative effect on placenta cellular processes; further studies are required.

## 5. Conclusions

**Figure 8:**
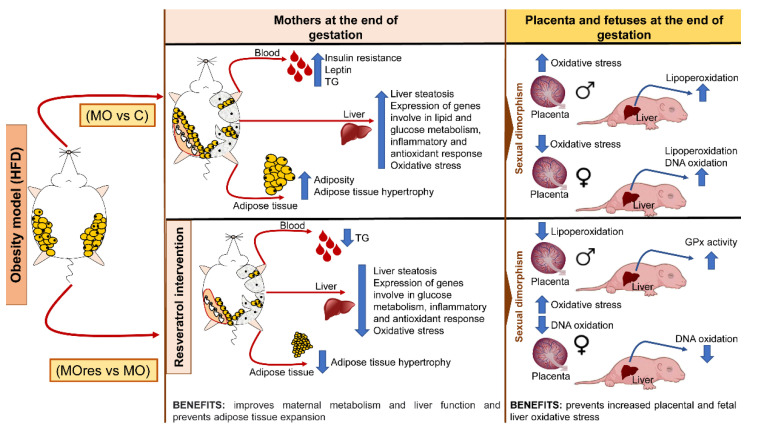
Summary of findings of the benefits of maternal resveratrol supplementation before pregnancy until the end of lactation.

Maternal obesity has a significant impact on the intrauterine environment and, consequently, on fetal development. Resveratrol has pleiotropic positive effects, including a potential protective impact against metabolic programming. In the present study, we show that at the dose used, resveratrol supplementation in obese mothers fed a high-fat diet improves insulin secretion, decreases adipocyte hypertrophy, liver steatosis, oxidative stress biomarkers, and reduces the expression of genes related to lipogenesis, insulin resistance, anti-inflammatory response, and the reduction of a transcription factor that is essential for the maintenance of a redox state. In addition, we found that maternal obesity increases placental and fetal liver oxidative stress in a sex-dependent manner, and that the effects of resveratrol supplementation also differ by gender (Figure 8). Our findings suggest that at the dose used, resveratrol supplementation prior to and during pregnancy did not have any negative effects and could be explored as an alternative therapy to improve obese mothers’ health and prevent the onset of metabolic diseases in their offspring in adult life.

## Figures and Tables

**Table 1:** Primers used in reverse transcription real-time quantitative PCR (RT-qPCR).

Accession Number	Gene	Sequence
NM_001276707	Sterol Regulatory Element-Binding Transcription Factor 1 (SREBP-1)	F: 5′-TGCGCAAGACAGCAGATTTA-3′ R: 5′-ACAAGATTGTGGAGCTCAAGG-3′
NM_017332	Fatty acid synthase (FAS)	F: 5′-GGCCACCTCAGTCCTGTTAT-3′ R: 5′-AGGGTCCAGCTAGAGGGTACA-3′
NM_001034925	Carnitine palmitoyltransferase 1C (CPT-1c)	F: 5′-TGTCCACAATTACCCGGATT-3′ R: 5′-GACGCCATACCCATGGTC-3′
NM_001168633	Insulin receptor substrate 2 (IRS-2)	F: 5′-CCAGGCACTGGAGCCTTA-3′ R: 5′-GCCCGCAGCACTTTACTC-3′
NM_017006	Glucose-6-phosphate dehydrogenase (G6PDH)	F: 5′-TTATCATCATGGGTGCATCG-3′ R: 5′-AAGGTGTCTTCGGGTAGAAGG-3′
NM_001108377	Phosphoenolpyruvate carboxykinase 2 (PEPCK2)	F: 5′-CCGACTGCACTGGTTCCT-3′ R: 5′-TCAGCCTGTGCCAGCTAAG-3′
NM_012589	Interleukin 6 (IL-6)	F: 5′-CCACTGCCTTCCCTACTTCA-3′ R: 5′-CTGGTCTGTTGTGGGTGGTA-3′
NM_012675	Tumor necrosis factor-alpha (TNF-alpha)	F: 5′-CACTTGGCTGAGAGGAAAGG-3′ R: 5′-CAAATGAGTGTCCCGCAGA-3′
NM_012854	Interleukin 10 (IL-10)	F: 5′-CAGATTCCTTACTGCAGGACTTTA-3′ R: 5′-CAAATGCTCCTTGATTTCTGG-3′
NM_031789	Nuclear factor erythroid 2–related factor 2 (Nrf2)	F: 5′-CAACAGTATTTCTGCCGCTGT-3′ R: 5′-CACAGGGAGGACTTTGTGAGT-3′
NM_017050	Cu/Zn cytosolic superoxide dismutase-1 (SOD-1)	F: 5′-GGTCCAGCGGATGAAGAG-3′ R: 5′-GGACACATTGGCCACACC-3′
NM_030826	Glutathione peroxidase-1 (GPX-1)	F: 5′-CGACATCGAACCCGATATAGA-3′ R: 5′-ATGCCTTAGGGGTTGCTAGG-3′
NM_013226	Ribosomal protein L32 (Rn-L32)	F: 5′-CCGGAAGTTTCTGGTCCAC-3′ R: 5′-CAGCACAGTAAGATTTGTTGCAC-3′

**Table 2:** Maternal fat depots weight at 19dG.

	C	Cres	MO	MOres
Body weight (g)	452 ± 7	429 ± 8	537 ± 14 *	514 ± 16 *
Total fat (g)	20.3 ± 2.4	12.4 ± 1	56.5 ± 5.5 *	50.7 ± 4.9 *
Adiposity index	4.5 ± 0.5	2.9 ± 0.3	10.5 ± 1 *	9.8 ± 0.9 *
**Fat depot (g)**				
Mediastinal	0.3 ± 0.07	0.3 ± 0.03	3.8 ± 2.7	1 ± 0.2
Retroperitoneal and perirenal	3.4 ± 0.5	2.7 ± 0.2	14.5 ± 1.2 *	14.1 ± 1.2 *
Omental	3.8 ± 0.5	3.9 ± 0.6	15.6 ± 3.2 *	13.6 ± 2.1 *
Parametrial and perivescical	4.3 ± 0.8	2.9 ± 0.2	11.9 ± 1.2 *	11.8 ± 1.3 *
Periovarian	2.8 ± 0.3	2.6 ± 0.3	10.8 ± 0.6 *	10.1 ± 0.8 *

Mean ± SEM, (C: control, n = 8; Cres: control + resveratrol, n = 10; MO: maternal obesity, n = 7; MOres: maternal obesity + resveratrol, n =7), * *p* < 0.05 vs their respective control. dG: days of gestation. No differences were observed between C and Cres and MO and MOres, and no interactions were observed between maternal diet and resveratrol supplementation.

## Data Availability

Data are contained within the article.
